# Restoration of miRNA-143 Expression Inhibits Growth and Migration of MKN-45 Gastric Cancer Cell Line

**DOI:** 10.34172/apb.2022.020

**Published:** 2020-10-20

**Authors:** Nayer Hosseinahli, Tahereh Zeinali, Nasrin Hosseinahli, Leila Karimi, Dariush Shanehbandi, Behzad Mansoori, Ali Mohammadi, Tohid Kazemi, Khalil Hajiasgharzadeh, Behzad Baradaran

**Affiliations:** ^1^Immunology Research Center, Tabriz University of Medical Sciences, Tabriz, Iran.; ^2^Gastrointestinal and Liver Diseases Research Center, Guilan University of Medical Sciences, Rasht, Iran.; ^3^Azarbaijan Higher Education and Research Complex, Tabriz, Iran.; ^4^Student Research Committee, Tabriz University of Medical Sciences, Tabriz, Iran.; ^5^Department of Immunology, Tabriz University of Medical Sciences, Tabriz, Iran.; ^6^Connective Tissue Diseases Research Center, Tabriz University of Medical Sciences, Tabriz, Iran.

**Keywords:** Gastric Cancer, miR-143, Replacement Therapy, Proliferation, Apoptosis, Migration Ability

## Abstract

***Purpose:*** Gastric cancer (GC) is one of the main causes of death from diseases, especially in developing countries. MicroRNAs (miRNAs) are important modulators of the messenger RNAs expression. Among these miRNAs, MiR-143 is a tumor suppressor miRNA and its irregular expression has been revealed in a diversity of malignancies such as GC.

***Methods:*** In this study, we have attempted to restore the miR-143 expression in MKN-45 cells by introducing pCMV-miR-143 plasmid vectors. The consequences of exogenous expression of miR-143 on cell proliferation and migration were assessed by MTT and scratch tests, respectively. In addition, the DAPI staining assay was applied for apoptosisquantification. Following miR-143 transfection, the changes in K-Ras, C-Myc, MMP9, Bax, Caspase-3, and Caspase-9 mRNA levels were assessed.

***Results:*** The results indicated that the enhanced expression of miR-143 had negative effects on MKN-45 cells proliferation and invasion. Moreover, decreased expressions of K-Ras, MMP9, and C-Myc and up-regulation of Bax, Caspase-3, and Caspase-9 as downstream targets of miR-143 were recognized.

***Conclusion:*** These experimental results indicate that reversing the miR-143 expression, by novel techniques, including miRNA replacement could be considered as an efficient approach to reduce cell survival and metastasis.

## Introduction

Gastric cancer (GC) is amongst the major causes of cancer-associated mortality worldwide.^1^ Despite initial response to conventional treatments such as chemotherapy and radiotherapy, drug resistance and disease relapse occur in a significant proportion of patients. Hence, developing new therapeutic methods, which can preferably reverse the disease state to normal, is one of the high priorities.^2^ In addition to environmental factors, GC is a result of the dysregulation of multiple genes.^3-6^ MicroRNAs (miRNAs) are small non-coding RNAs that modulate post-transcriptionally the expression of their target genes. This happens by base pairing to the targeted mRNA and involvement of the relating defense system.^7^ Impaired expression of miRNAs in different cancers is reported frequently. MiR-143 is one of the anti-oncogenic miRNAs which its downregulation is evident in a wide range of human malignancies.^8^ This miRNA has a preventative role in tumor development and modulates the expression of multiple genes such as K-Ras, Bcl2, DNMT3A, ERK5, MYO6, Bax, caspase-3, caspase-9, and ELK1 that are included in cell growth, survival, differentiation, and invasion.^7,9^ Due to several regulatory roles, restoration of miR-143 expression seems to be an efficient approach in the treatment of GC.

In the current study, a plasmid vector coding for miR-143 was transfected into the MKN-45 cell line. Quantitative real-time polymerase chain reaction (qRT-PCR) was used after transfecting cells with miR-143 to determine the optimum dosage based on the expression of this miRNA. After restoring the expression of miR-143, the consequent impacts on cell survival, apoptosis, and migration were determined by relative assays. In this regard, the MTT assay was used to evaluate the viability of MKN-45 cells after miR-143 restoration. Wound healing assay was used to observe migration in MKN-45 cell line and also, the effect of miR-143 on the migration rate of the cells. Moreover, qRT-PCR was used as a method to determine the effect of miR-143 on the expression of K-Ras, C-Myc, MMP9, Bax, caspase-3, and caspase-9 genes, which are known to be involved in apoptosis, invasion and migration of MKN-45 cell lines in GC.

## Materials and Methods

### Cell culture

The human gastric cancer cell line (MKN-45) was purchased from the Pasteur Institute of Iran and cultured in RPMI medium supplemented with 10% FBS and penicillin/streptomycin mixtures (Gibco, Carlsbad, CA, USA). The rest of the materials were bought from Santa Cruz Biotechnology (Santa Cruz, CA, USA), unless otherwise specified in the text. The cells were grown at 37°C incubator in a humidified atmosphere of 5% CO_2_ and were used in the logarithmic phase of growth according to our previous studies.^10^

### Plasmid vector amplification

The pCMV-miR-143 vector, expressing miR-143 precursor, was purchased from OriGene Company (Rockville, MD, USA). Along with the mentioned vector, an empty pCMV vector was considered as blank control. Amplification of the plasmid vectors was carried out by molecular cloning in *E. coli* (DH5α) bacteria.^11^ Resistance to kanamycin was utilized as a selection marker and the large-scale plasmid DNA extraction from the transformed bacteria was performed using the Maxi- Prep kit (Yekta Tajhiz, Tehran, Iran). Moreover, the analysis of the vectors was conducted using a NanoDrop device (Thermo Scientific, USA).

### Transfection of the gastric adenocarcinoma cells

5×10^5^ MKN-45 cells were cultured in a 6 well plate. Distinct wells were considered for pCMV-miR-143 transfected cells (vec + ) and the cells transfected with the empty pCMV vector (vec-). After 24 hours, the cells were checked for proper adhesion and confluence. After reaching ~60% confluence, 6 μg of each plasmid vector (OriGene, Rockville, MD) was utilized for the transfection of MKN-45 cells using jetPRIME® transfection reagent (PolyPlus, France). In brief, the plasmid vectors were diluted in 150 mM NaCl to a total volume of 200 μL in a 0.5 mL microtube. In another 0.5 mL microtube, 6 μL of jetPRIME® reagent was diluted in 194 μL of 150 mM NaCl. Subsequently, the contents of the mentioned reaction tubes were mixed and kept for 30 minutes at 25°C in the darkroom. The mixture was added to the cultured cells drop-wise. After 24 hours, the cells were monitored for successful transfection. The utilized vectors encode a green fluorescent protein (GFP) for transfection monitoring. Cytation^TM^ 5, Cell Imaging Multi-Mode Reader System (Cytation 5, Biotek, Winooski, VT) was employed to detect the GFP expressing cells. 48 hours after transfection, Geneticin (G418, Gibco) treatment (4 μg/μL), was started for the selection of the cells with stable miRNA expression. Also, the ratio of stably transfected cells was evaluated by separate flow cytometry analysis (MACS-Quant 10, Miltenyi Biotec, Germany). Briefly, the transfected cells emitted the green fluorescence because of GFP expression so the percentage of GFP positive cells evaluated in the FITC channel.

### Relative quantification of miR-143 expression in the cells with stable transfection

Quantitative real-time PCR was applied to confirm the successful restoration of miR-143 expression in the stably transfected MKN-45 cells (cells transfected with vec- were considered as control). Total RNA was extracted using RiboEx reagent (GeneAll, Korea) from the cells treated with Geneticin for 2 weeks. Synthesis of cDNA for miR-143 quantification was carried out using a cDNA synthesis kit (Exiqon, Denmark); therefore, 10 ng of total RNA was utilized for this purpose. Then, the qRT-PCR was applied at a total volume of 10 μL using 5 μL of 2X SYBR green premix (Takara, RR820L), 4 μL of 1:80 diluted cDNA, and 1 μL of a specific primer for miR-143 (Exiqon, Denmark) on a Light Cycler 96 system (Roche Diagnostics, Mannheim, Germany). PCR included an initial hot start step at 94°C followed by 45 cycles of 94°C for 10 seconds and 60°C for 1 minute and reported by the Livak method.^12^ In addition, the miR-103 served as a normalizer group.^8^

### Effect of miR-143 restoration on cell viability

The effects of miR-143 replacement on the viability of the MKN-45 cells were assessed by MTT assay. For this purpose, 8×10^3^ cells (vec- and vec + cells) were cultured in 96-well plates in triplicates. Then, 4 days later, the cells were treated with 50 μL of MTT solution (2 mg/mL; Lot no. DU21373R2; Bio Basic, Canada) at 37°C for 4 h. Then, 200 ml of dimethyl sulfoxide was used to dissolve the resulting formazan.^13^ After incubation at 37°C for 30 minutes, absorbance was estimated at a test wavelength of 570 nm and a reference wavelength of 620 nm. using a Sunrise^TM^ microplate reader (Tecan, Switzerland).

### Assessment of in vitro cell migration

Wound healing (Scratch) assay was used to study the effects of miR-143 transfection on MKN-45 cells migration.^14^ The GC cells were cultured to 80% confluence in 24-well plates Subsequently, the cell layer was scratched with a sterile yellow pipette tip. The migratory ability of the miR-143 transfected cells was monitored from 0 to 96 hours with a microscope (Optika, Italy) and compared to the vec- transfected cells.

### Apoptosis detection by DAPI staining

4,6-Diamidino-2-phenylindole(DAPI) staining for apoptotic cell detection was done as previously explained.^15^ This method is based on fluorescence creation following the binding of DAPI to DNA molecules. Vec + and vec- transfected cells were seeded in 6-well plates. Following 24 h, cells were fixed using paraformaldehyde (4%). After 15 min, cells were rinsed with PBS and permeabilized using 0.1 % Triton-X-100 for 10 minutes. DAPI in a concentration of 1:500 (in PBS) was utilized for staining. Then, 10 minutes later, depending on the morphological features of the nuclei, cells were recognized as viable or apoptotic cells with fragmented nuclei. Citation 5 cell imaging system (CYTATION5; Biotek, Winooski, VT) was employed for imaging.

### Relative quantification of miR-143 putative targets

Alterations in the expression of MMP9, K-Ras, C-Myc, Bax, caspase-3, and caspase-9 genes as putative targets of miR-143 were quantified by qRT-PCR. 3 μg of the extracted RNA was used for cDNA synthesis by random hexamer primer and RevertAid^TM^ Reverse Transcriptase (RT) (Thermo Fisher Scientific). QRT-PCR was done in a final volume of 10 μL (5 μL of 2X SYBR green premix, 0.2 μL of 4 μM primers and 0.5 μL of cDNA) on a Light Cycler 96 system. The cycling program was composed of an initial hot start step at 94°C followed by 45 cycles of 94°C for 10 seconds, 59°C for 30 seconds and 72°C for 20 seconds. Relative expression was calculated by the Livak method^12^ and β-actin was employed as the housekeeping gene. Primer sequences for the analyzed genes were designed by Primer Blast online and the sequences are listed in Table 1.

**Table 1:** Specifications of the primers used for qRT-PCR

**Genes**		**Sequences**	**Product Size**
K-Ras	Forward	5´ CTCCCTGTGTCAGACTGCTCTTT 3´	154 bp
	Reverse	5´ GGCCTTGCAACCTTGGTCTCTTC 3´	
MMP9	Forward	5´ GGTTCTTCTGCGCTACTGCTG 3´	187 bp
	Reverse	5´ GTCGTAGGGCTGCTGGAAGG 3´	
C-Myc	Forward	5´ AGGCTCTCCTTGCAGCTGCT 3´	163 bp
	Reverse	5´ AAGTTCTCCTCCTCGTCGCA 3´	
Caspase-3	Forward	5´ TGTCATCTCGCTCTGGTACG 3´	120 bp
	Reverse	5´ AAATGACCCCTTCATCACCA 3´	
Caspase-9	Forward	5´ GCAGGCTCTGGATCTCGGC 3´	152 bp
	Reverse	5´ GCTGCTTGCCTGTTAGTTCGC 3´	
Bax	Forward	5´ TTTGCTTCAGGGTTTCATCCA 3´	151 bp
	Reverse	5´ CTCCATGTTACTGTCCAGTTCGT 3´	
β-actin	Forward	5´ TCCCTGGAGAAGAGCTACG3 3´	131 bp
	Reverse	5´ GTAGTTTCGTGGATGCCACA 3´	

### Statistical analyses

Statistical analysis was performed using GraphPad Prism 6 software (San Diego, CA, USA). Data were expressed as mean ± standard errors of the mean. For two groups student’s *t* test and in group comparisons, one-way ANOVA followed by Bonferroni post-test analyses was used. *P* < 0.05 was considered significant.

## Results

### Confirmation of the transfection of MKN-45 cells

**Figure 1:**
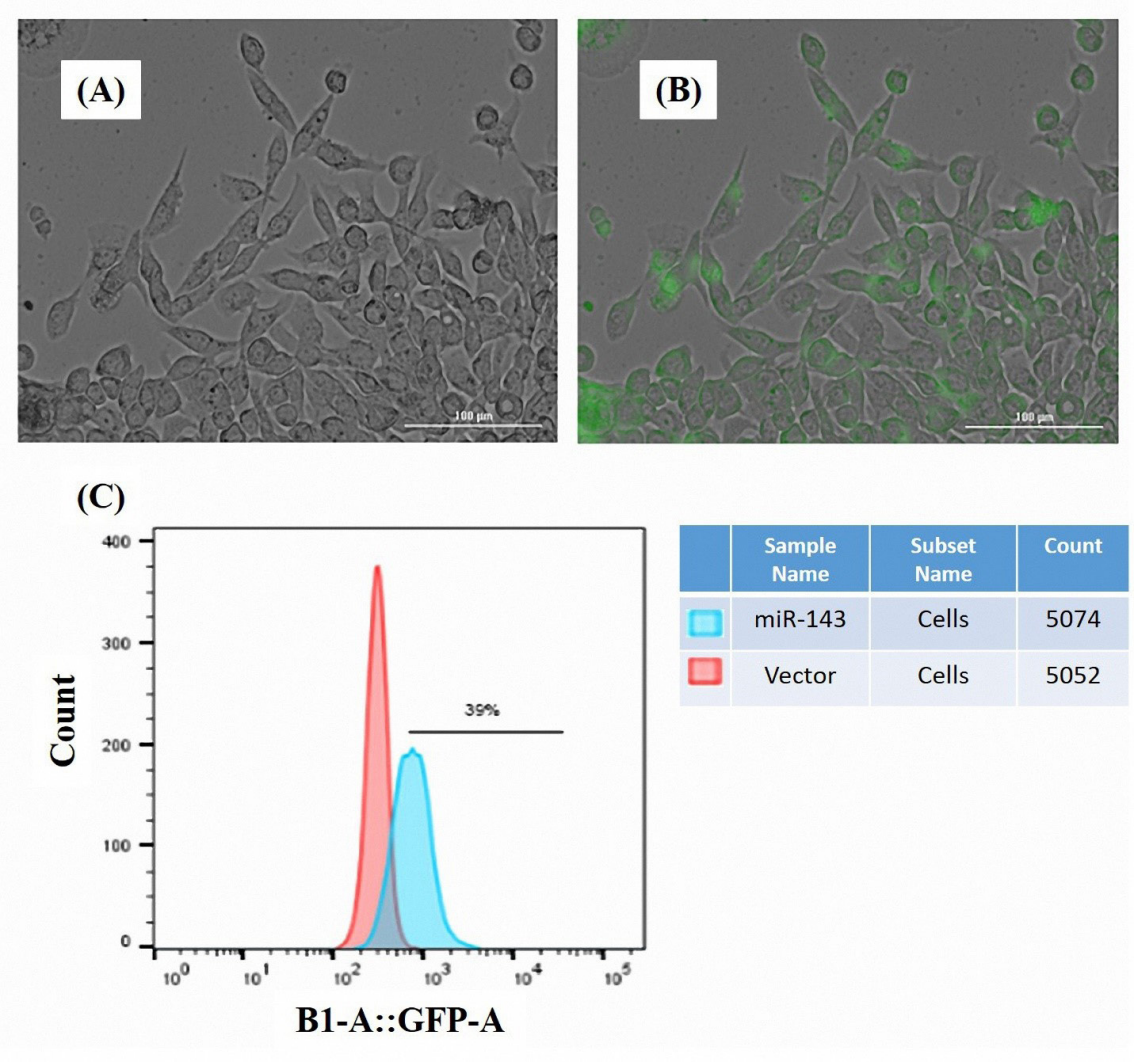
Assessment of the transfection of the MKN-45 cells. Expression of GFP marker was detected by Cytation 5 imaging system indicating a high-efficiency transfection of the MKN-45 cells (A,B) cells observed by a bright field filter, (A) the same cells were observed by GFP filter, (B). Flow cytometry was performed to quantify the expression level of GFP in the transfected cells. The results revealed an increased GFP gene expression in the miR-143 transfected cells (C).

24 h after transfection, the cells were monitored for the successful delivery of the plasmid vectors. Cytation^TM^ 5 system was used for the detection of GFP expression in the transfected cells. Figure 1A and 1B indicate the fluorescence in the MKN-45 cells transfected with miR-143 vector. The findings of flow cytometry analysis revealed that GFP was overexpressed in the miR-143-transfected MKN-45 cells (Figure 1C).

### Quantification of miR-143 expression in the transfected MKN-45 cells

**Figure 2:**
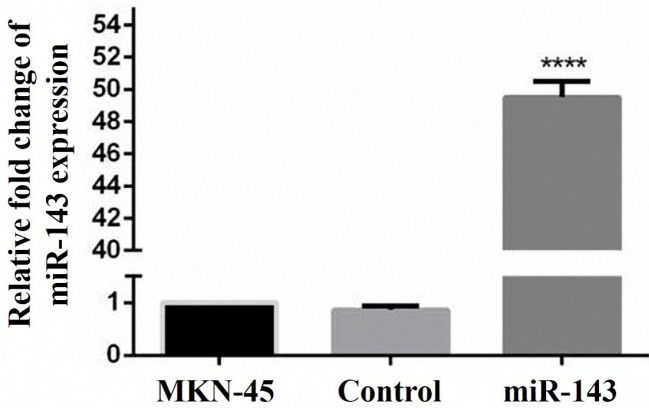
Restoration of miR-143 expression was confirmed by the qRT-PCR. The up-regulation of miR-143 in the stably transfected cells confirmed a successful miRNA transfection. Approximately, a 50-fold increase in miR-143 expression was evident in transfected cells (****P < 0.0001).

Geneticin (G418) treatment was employed for the screening of the cells stably expressing miR-143. Two weeks after transfection, qRT-PCR was applied to assess the expression level of miR-143 in the transfected and control cells. The results designated the enhanced miR-143 expression in comparison to the controls transfected with the empty vector (Figure 2).

### Effect of miR-143 replacement on cell proliferation

**Figure 3:**
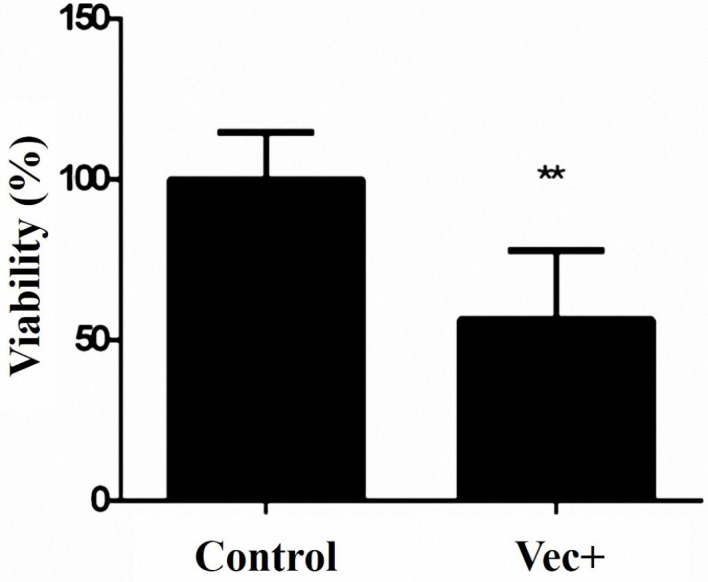
The cytotoxic effect of miR-143 restoration on MKN-45 cells proliferation. The viability of the transfected cells with pCMV-miR-143 has been lowered to ~57% compared with the control group (**P < 0.01).

MTT assay was applied for the evaluation of cell viability following miR-143 transfection. As shown in Figure 3, miR-143 overexpression significantly lowered the viability of the MKN-45 cells in comparison to the cells receiving vec- (control).

### Impacts of miR-143 replacement on the migration of MKN-45 cells

**Figure 4:**
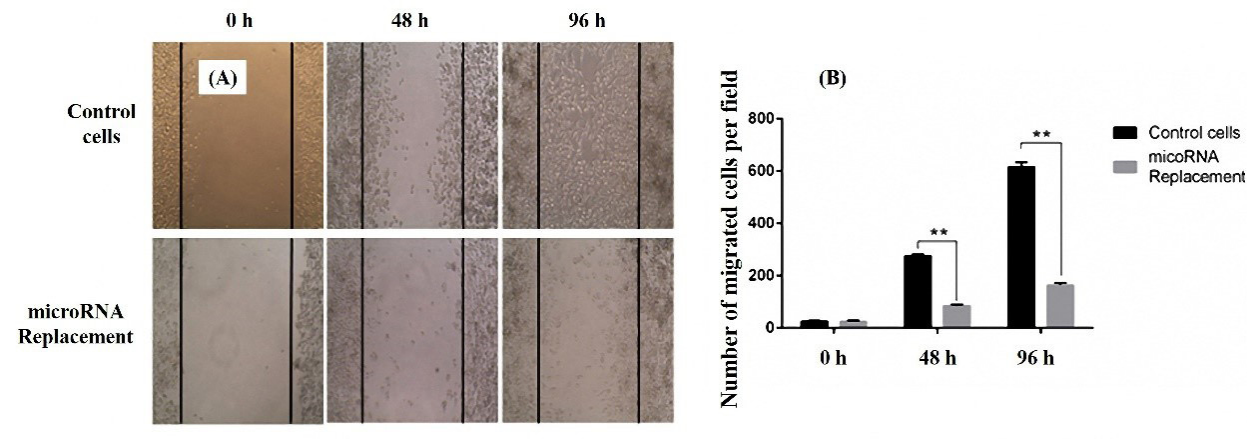
Wound healing assay for evaluation of migration in miR-143 transfected MKN-45 cells. The number of migrated cells in both 48 and 96 h are considerably low in stably expressing miR-143 cells (A). The same results are shown in the graph format (B). Data are presented as mean ± SEM. (n = 3; **P < 0.01).

Scratch test was utilized to evaluate the effects of miR-143 transfection on MKN-45 cells migration. Microscopic imaging was applied to document the migration events. MKN-45 cells treated with Pcmv-miR-143 vector or control (vec−) were analyzed according to amount of the migrated cells. The results indicated that the cells with increased miR-143 expression had a significant loss in the migration rate in both 48 and 96 hours (Figure 4). This investigation designates that miR-143 may have a negative effect on GC cells migration.

### Assessment of apoptosis occurrence in miR-143 transfected cells

**Figure 5:**
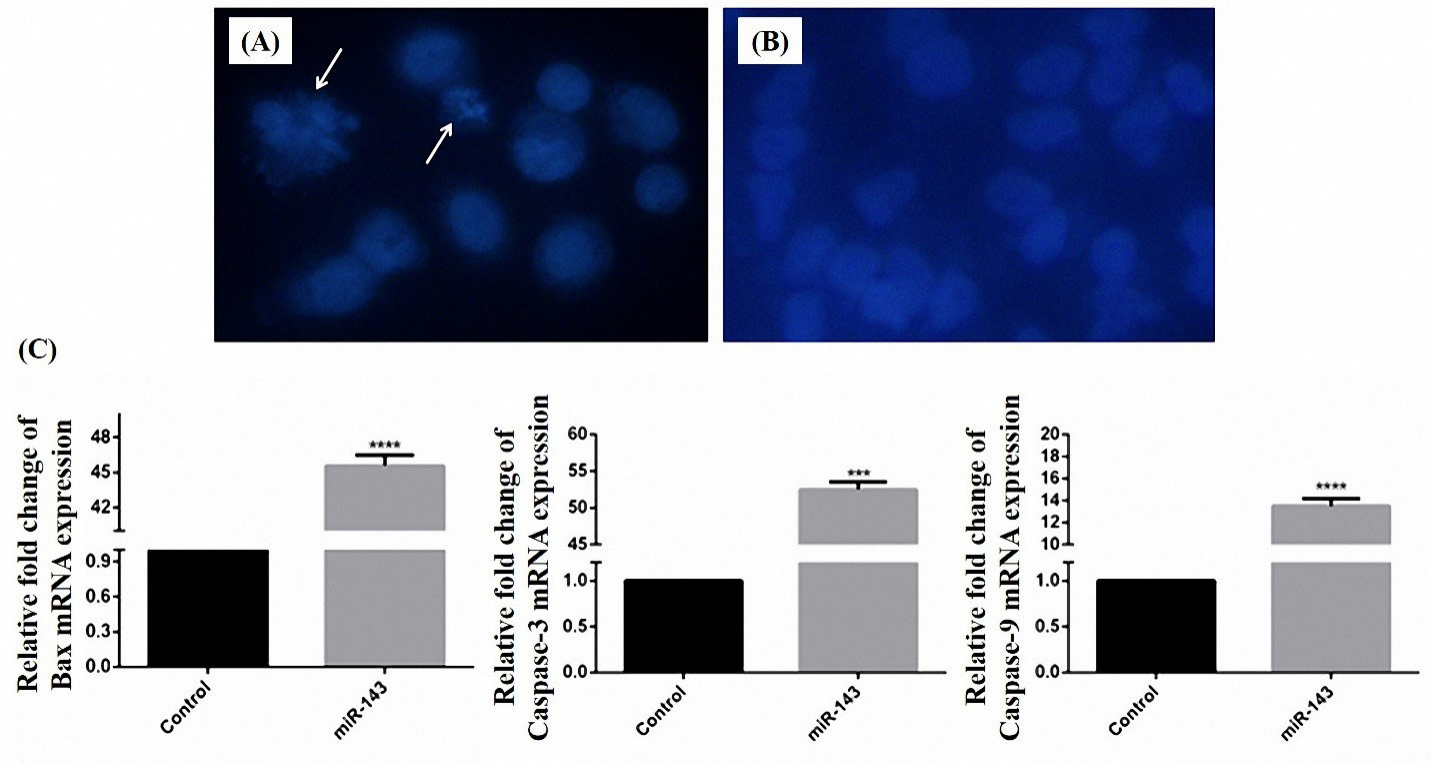
DAPI staining for detection of apoptosis in MKN-45 cells transfected with vec + (A) and vec- (B). Rate of apoptosis according to the percentage of the cells with fragmented nuclei that pointed out by arrows. The mRNA expression level of Bax, caspase-3, and caspase-9 increase 45.55, 52.48, 13.48 fold after replacement of miR-143 (C). (**** *P* < 0.0001 and *** *P* < 0.001).

DAPI staining was performed for apoptosis quantification in miR-143 transfected cells. The results of the apoptosis assay showed that condensed or fragmented chromatins decreased in miR-143 transfected cells in comparison with non-treated cells (Figure 5). These condensed chromatins in DAPI staining experiments were considered as apoptotic nuclei.

### Evaluation of miR-143 target genes expression

**Figure 6:**
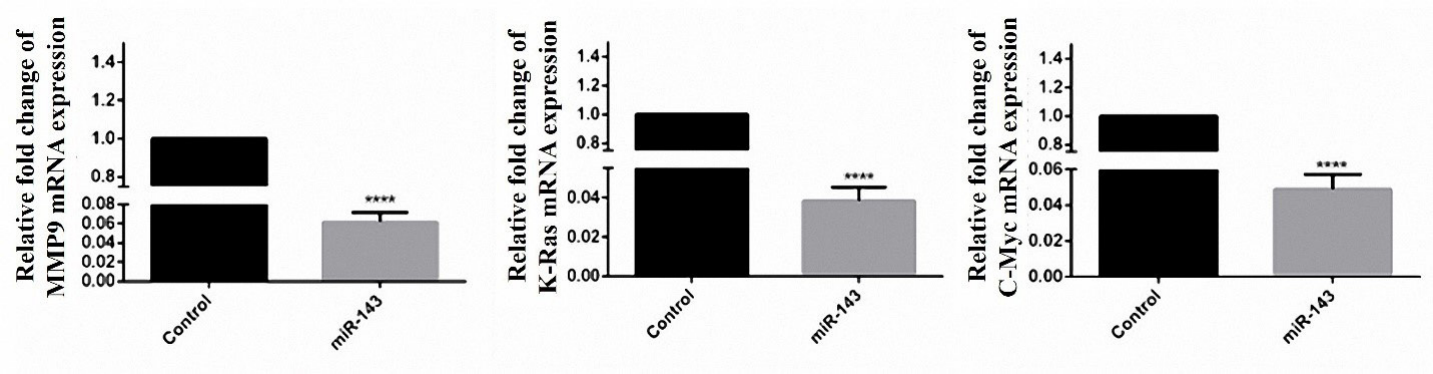
Effect of miR-143 restoration on C-Myc, K-Ras, and MMP9 expression in mRNA level. Levels of C-Myc, K-Ras, and MMP9 mRNAs were significantly decreased in cells with restored miR-143 expression compared with the control. Cells transfected with an empty pCMV vector were considered as control. Data are presented as mean ± SEM. (****P < 0.0001).

Variations in MMP9, K-Ras, and C-Myc mRNAs were evaluated in stably miR-143 expressing cells. According to the findings, there was a significant decrease in mRNA levels of the mentioned genes compared to the vec- transfected cells. The expression ratio of MMP9, K-Ras, and C-Myc genes was lowered to 16.66, 26.31 and 20.83 fold respectively (Figure 6).

## Discussion

Dysregulation of miRNA expression is frequently reported in a diversity of human cancers. This accounts for tumor suppressor miRNAs which are best known for targeting genes with oncogenic properties.^16,17^ Consequently, the restoration of onco-suppressor miRNAs with downregulated expression seems to be a promising approach in fighting cancer. MiRNA based therapeutics in human malignancies aim to interfere with different aspects of cancer including tumorigenesis, angiogenesis, epithelial-mesenchymal transition and metastasis.^16,18^ MiRNA replacement therapy is an example of such interventions with promising outcomes. They showed that restoration of let-7 expression can restrain lung cancer both *in vitro*and in an animal model.^19^ MiRNA replacement has recently attracted more interest compared to conventional gene therapy. These miRNAs can target different genes and pathways and the regulation of a single miRNA can be more advantageous. Nevertheless, there is no comprehensive data about the exact targets of miRNAs yet and selecting a miRNA for manipulation should be done prudently.^20^ Takagi and colleagues in 2008 assessed the expression of miR-143 in cancerous tissues of 43 patients with gastric neoplasm. They also studied the expression rates of this miRNA in various GC cell lines *in vitro*. A considerable decrease of miR-143 expression was evident in all cases and the MKN-45 cell line had the most significant reduction.^21,22^ In this study, miR-143 was chosen as a candidate for replacement therapy. The importance of miR-143 due to its involvement in different aspects of cancer has been reviewed previously.^23-25^ According to the literature, the downregulation of miR-143 is correlated with cancer growth, apoptosis, and metastasis.^26,27^ These events could be potentially due to the lack of regulatory effects of miR-143 on K-Ras, C-Myc, two matrix metalloproteinase genes (MMP9 and MMP13), Bax, caspase-3, and caspase-9.^6,28-32^ K-Ras is an important signaling molecule in viable cells. However, K-Ras mutants play a pivotal function in the progression of cancerous cells. Moreover, the C-Myc transcription factor is a pivotal regulator of cell proliferation and possesses clinical significance in different types of malignancies such as lung, pancreas, and colorectal cancers.^33-35^

According to the results of this study, the inhibitory effect on proliferation was observed because of miR-143 overexpression in the target cells. This may occur in part due to the suppression of K-Ras and C-Myc genes. Since, following the exogenous expression of miR-143 in MKN-45 cells, K-Ras and C-Myc genes were significantly downregulated. Chen et al have also investigated the impacts of miR-143 restoration on the proliferation of cancerous cells. They utilized synthetic RNA oligonucleotides (mimicking miR-143 precursors) for neutralizing K-Ras mRNA in colorectal cancer cells and indicated that the proliferative potential of “Lovo” colorectal adenocarcinoma cells was lowered.^36^ Inhibitory effect of miR-143 expression and its downstream target (C-Myc) has been described previously in colorectal cancer^37^ and B-cell lymphoma.^38^ In addition to the oligonucleotide mimics, vectors through the increased expression of the target miRNA are introduced for miRNA restoration. In a study, Tavanafar et al utilized plasmid vectors to perform the miR-143 replacement in the breast cancer cells line and recognized a reduction in K-Ras expression and cell growth.^8^ They also performed a decreased expression of metastasis-related genes including Vimentin, CXCR4, and MMP9. On the other hand, the involvement of miRNAs in epithelial-mesenchymal transition has been shown in different metastatic cell lines.^39^ On the other hand, the matrix metalloproteinase proteins are engaged in the breakdown of the extracellular matrix and facilitate cell migration and metastasis. Huang et al indicated that miR-143 and miR-145 have central roles in modulating bone metastasis of prostate cancer cells via inhibiting some cancer markers such as C-Myc, CD133, Klf4, CD44 and Oct4.^40^ Overexpression of miR-143 has been reported to block the metastasis of pancreatic cancer via decreasing the MMP9 protein.^41^ Last but not least, cell migration was significantly declined after miR-143 introduction in our study. The decreased levels of MMP9 mRNA after this intervention, suggests an anti-metastatic effect for miR-143 restoration. Moreover, in this study, we assessed apoptosis by the DAPI staining and expression analysis of Bax, caspase-3, and caspase-9 genes. Increased levels of pro-apoptotic Bax, caspase-3, and caspase-9 lead to loss of mitochondrial membrane potential that is a critical process in the initiation of apoptosis.^31,42,43^ The results of DAPI staining demonstrated a significant increment in apoptosis for the miR-143 receiving cells in comparison to the control group. Furthermore, after restoring miR-143 expression in the MKN-45 cells, the expression of Bax, caspase-3, and caspase-9 increased by 45.55, 52.48, 13.48 fold respectively. This is in line with the study conducted by Pedro and colleagues on colon cancer in 2009. They observed that introducing miR-143 mimics leads to increased expression of caspase-9 and caspase-3 genes and apoptosis by 60% in the transfected cells. In 2016, three independent studies on osteosarcoma, prostate and cervical cancers, showed increased expression of miR-143 results in an increase of 45%-60% in apoptosis.^21,26,44,45^

## Conclusion

**Figure 7:**
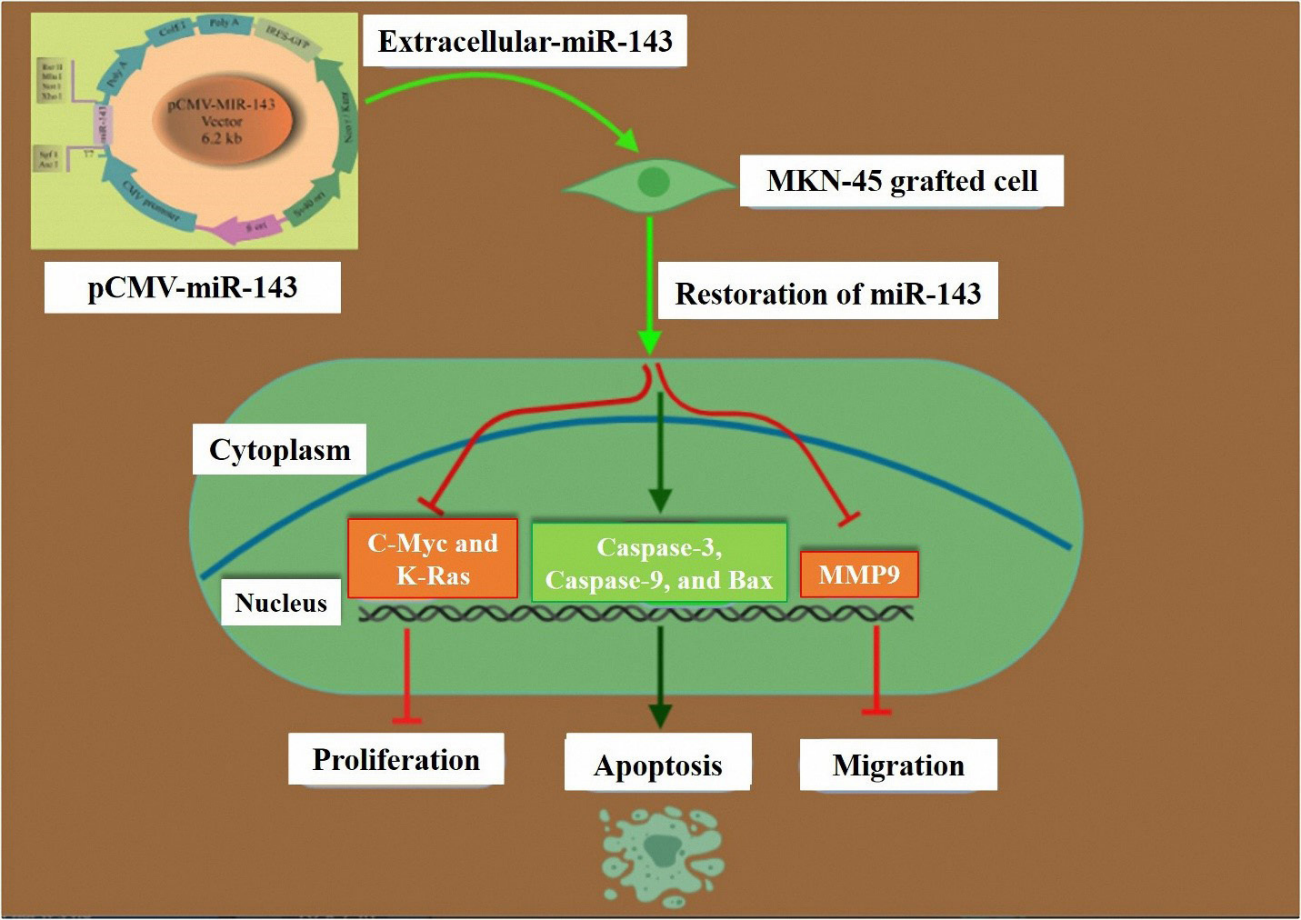
Schematic representation of the impact of miR-143 restoration on proliferation, apoptosis, and migration of GC cells. PCMV-miR-143 increases the gene expression of the miR- 143 in transfected MKN-45 cells. Restoration of miR-143 suppresses c-Myc, K-Ras, MMP-9. Restoration of miR-143 stimulates the caspase-3, caspase-9, and Bax in the miR-143 transfected cells.

Restoration of miR-143 expression by plasmid vector transfection resulted in decreased proliferation and migration in the MKN-45 cell line. Expression of K-Ras, MMP9, and C-Myc as putative targets of miR-143 was lowered in the transfected cells (Figure 7). Furthermore, the miR-145 expression is able to cause the apoptosis of GC MKN-45 cells possibly by increasing the expression of Bax, caspase-3, and caspase-9. Restoration of miR-143 expression by such novel approaches including miRNA replacement could be efficient in the treatment of GC.

## Ethical Issues

All experiments and procedures were conducted in compliance with the ethical principles of Tabriz University of Medical Science, Tabriz, Iran and approved by the regional ethical committee for medical research (Ethical code: TBZMED.REC.1394.792).

## Conflict of Interest

The authors have no conflicts of interest to declare.

## Acknowledgments

The authors would like to thank the Immunology Research Center, Tabriz University of Medical Sciences for their support.
